# Optogenetic Therapy for Visual Restoration

**DOI:** 10.3390/ijms232315041

**Published:** 2022-11-30

**Authors:** Daiki Sakai, Hiroshi Tomita, Akiko Maeda

**Affiliations:** 1 Department of Ophthalmology, Kobe City Eye Hospital, Kobe 650-0047, Japan; 2 Department of Ophthalmology, Kobe City Medical Center General Hospital, Kobe 650-0047, Japan; 3 Department of Surgery, Division of Ophthalmology, Kobe University Graduate School of Medicine, Kobe 650-0017, Japan; 4 Graduate Course in Biological Sciences, Division of Science and Engineering, Iwate University, Iwate 020-8550, Japan

**Keywords:** optogenetics, gene therapy, retinitis pigmentosa, retina, retinal degeneration, retinal dystrophy, blindness, visual restoration, channelrhodopsin, opsin

## Abstract

Optogenetics is a recent breakthrough in neuroscience, and one of the most promising applications is the treatment of retinal degenerative diseases. Multiple clinical trials are currently ongoing, less than a decade after the first attempt at visual restoration using optogenetics. Optogenetic therapy has great value in providing hope for visual restoration in late-stage retinal degeneration, regardless of the genotype. This alternative gene therapy consists of multiple elements including the choice of target retinal cells, optogenetic tools, and gene delivery systems. Currently, there are various options for each element, all of which have been developed as a product of technological success. In particular, the performance of optogenetic tools in terms of light and wavelength sensitivity have been improved by engineering microbial opsins and applying human opsins. To provide better post-treatment vision, the optimal choice of optogenetic tools and effective gene delivery to retinal cells is necessary. In this review, we provide an overview of the advancements in optogenetic therapy for visual restoration, focusing on available options for optogenetic tools and gene delivery methods.

## 1. Introduction

### 1.1. Retinal Structure and Visual Pathway

**Figure 1:**
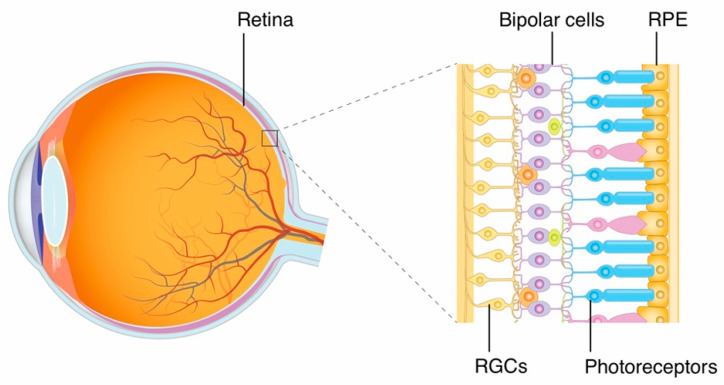
The retina is a layered structure lining the back of the eyeball. The visual pathway in the retina consists of three neurons: photoreceptors, bipolar cells, and retinal ganglion cells (RGCs). The retinal pigment epithelium (RPE) maintains retinal function.

The neural retina has a layered structure consisting of five different types of cells, including photoreceptors, bipolar cells, retinal ganglion cells (RGCs), horizontal cells, and amacrine cells. The retinal pigment epithelium (RPE), located on the outermost side of the retina, maintains retinal function. The retinal vessels, which supply and drain the retina, consist of retinal endothelial cells and pericytes. Among these cells, only the photoreceptors respond to light to provide vision. As first-order neurons in the visual pathway, photoreceptors initiate the transduction process of visual signals. Then, visual signals are transmitted to the second-order neurons of bipolar cells, and subsequently to the third-order neurons of RGCs, while horizontal cells and amacrine cells are considered to facilitate the integration of visual signals (Figure 1).

### 1.2. Optogenetics and Retinal Degenerative Diseases

**Figure 2:**
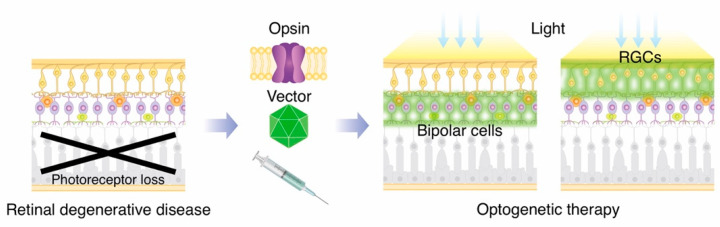
Basic concept of optogenetic therapy for visual restoration. Photoreceptors are lost in eyes with retinal generative diseases. Using gene transfer techniques, remaining retinal cells such as bipolar cells or retinal ganglion cells (RGCs) are converted into artificial photoreceptors.

Optogenetics is a breakthrough technology in neuroscience that enables the control of neural activity through ectopic expression of light-activated optogenetic proteins in genetically modified target cells [1]. One of the most promising applications of optogenetics is in the treatment of retinal degenerative diseases. Retinitis pigmentosa (RP) is a representative hereditary retinal degenerative disease with a worldwide prevalence of approximately 1 in 4000 individuals, making it the most common cause of blindness in developed countries [2]. RP encompasses a genetically heterogeneous group of retinal degenerative diseases. More than 70 causative genes have been identified to date (RetNet; https://sph.uth.edu/retnet/ accessed on 29 September 2022.); however, patients with RP share a common pathophysiology of eventual photoreceptor cell death. It is known that the inner retinal layer, including bipolar cells and RGCs, is preserved for a certain period after the loss of photoreceptors in RP patients [3,4]. Thus, the inner retinal layer could be a target for optogenetic therapy. The basic concept of optogenetic therapy for retinal degenerative diseases is to convert the remaining non-light-sensitive retinal cells into artificial photoreceptors through the expression of light-activated proteins (Figure 2).

Recent research progress on the genetic aspect of RP has not only led to a deeper understanding of the molecular mechanism of retinal degeneration, but also advanced novel gene therapy [5]. Currently, the major strategy of retinal gene therapy is to compensate for loss of function by replacing a specific defective gene with a healthy one. Although novel retinal gene therapies are highly encouraging for patients with RP, which is currently considered an untreatable disease, this approach has some limitations. First, while gene-specific intervention such as gene supplementation therapy is an elegant solution for monogenic diseases, each treatment can only be applied to a limited population of patients with corresponding gene variants. Moreover, almost half of the patients who undergo genetic testing cannot reach genetic diagnosis [6,7]. Therefore, there is a high demand for gene-independent therapies. Second, gene supplementation therapy requires living photoreceptors for rescue, which is not applicable to advanced diseases with no remaining photoreceptors. A method to restore sight to blindness caused by the advanced diseases is definitely warranted. Optogenetic therapy, as an alternative gene therapy approach, can provide visual restoration for late-stage retinal degeneration, regardless of the genotype.

Bi et al. first reported in 2006 that the expression of channelrhodopsin-2 (ChR2) in RGCs restores light responses in mice with retinal degeneration [8]. Subsequently, many researchers have been improving this promising optogenetic therapy, and multiple clinical trials are currently ongoing, less than a decade since the first attempt. Successful translation of optogenetics from bench to clinic has been achieved based on accumulated experiences spanning several different fields, and cross-disciplinary knowledge is required to follow advancements in optogenetic therapy. In this manuscript, we aim to provide a concise overview by dissecting the elements of optogenetic therapy into strategies for selecting target cells, available optogenetic tools, and gene delivery methods.

## 2. Strategies and Target Cells

The components of the three-neuron chain in the retina—photoreceptors, bipolar cells, and RGCs—could be targets of optogenetic therapy and have been practically considered in previous studies.

### 2.1. Dormant Cone

Patients can take advantage of the innate signal-processing function of the retinal circuit by targeting upstream neurons. In patients with RP, rod photoreceptors degenerate primarily due to genetic defects, followed by the slow degeneration of cone photoreceptors. The mechanism of cone degeneration remains unclear; however, non-autonomous mechanisms including oxidative stress, trophic factors, metabolic stress, light damage, and inflammation are suggested [9]. Early changes in cone degeneration include shortening or loss of outer segments, while cell bodies remain intact [10]. These surviving cone photoreceptors without light sensitivity are called dormant cones, whose potential for functional rescue has attracted researchers’ interest [11]. Hyperpolarizing opsins are rational optogenetic tools that can resemble the trigger of the original phototransduction pathway. This proof-of-concept was demonstrated using the light-activated chloride pump halorhodopsin obtained from *Natronomonas pharaoni* (NpHR) [12] in mouse models of RP (*Cnga3*^−/−^; *Rho*^−/−^ double-knockout and *rd1* mice) [13]. Non-specific expression of hyperpolarizing opsins may have a negative impact on subsequent signal processing. Thus, target-specific delivery methods are preferred for this strategy. Although cone reactivation using a hyperpolarizing optogenetic tool is a promising option for restoring vision in patients with dormant cones, secondary and tertiary neurons need to be targeted in patients with late-stage RP with no residual primary neurons, photoreceptors.

### 2.2. Bipolar Cell

When the photoreceptors are completely lost, bipolar cells are candidate neurons to be targeted, which also benefit from the retinal circuit to some extent. Retinal processing, which augments the visual signal, could be preserved by targeting bipolar cells, which is expected to enhance the visual outcome. Lagali et al. first targeted ChR2 to ON bipolar cells using in vivo electroporation in *rd1* mice, and achieved the recovery of electrophysiological response and visually guided behavior [14]. This strategy has had difficulties in gene delivery because bipolar cells are isolated from both the vitreous and subretinal spaces. However, using modified viral vectors, subsequent studies successively demonstrated the recovery of visual function after ON bipolar cell-targeted optogenetic therapy in RP mouse models [15,16,17,18,19,20,21]. This strategy requires good condition of the inner retinal layer with intact synaptic connections between bipolar cells and RGCs. Visual function recovery might be deteriorated by inner retinal remodeling in the later stage of RP [22]. An important issue is the extent to which we can expect functional benefits from bipolar-targeted versus downstream RCG-targeted therapy. Some studies have described the advantages of bipolar-targeted therapy [23,24], but the opposite result has also been reported [25]. Verification of the theoretical priority provided by preserving retinal processing would be the key to developing bipolar cell-targeted optogenetic therapy. Additionally, gene delivery to primate bipolar cells seems more challenging than in rodents [26], which should be overcome.

### 2.3. RGC

RGC-targeted optogenetic therapy is a necessary option, representing the “final frontier,” because the innermost RGCs seem to be the most stable during the degeneration course [22]. Indeed, RGC-targeted optogenetic therapy is the most-studied strategy to date. Based on the evidence of successful functional recovery in animal experiments, preceding clinical trials adopted the RGC-targeting approach (Section 5). A case report in which patients with advanced RP showed partial recovery of visual function through RGC-targeted expression of ChrimsonR has already been published [27]. It is known that RGCs originally have diversity in features (at least 18 different cell types are found in the primate retina [28,29]), which contributes to complicated visual signal processing. Since the current trend in optogenetic therapy is non-selective activation of RGCs by ubiquitous promoters, the visual function brought by this approach must be understandably altered from the innate function. Noteworthy in this regard is how useful the visual function provided can be in daily life. Optogenetic therapy will undoubtedly be a substantial treatment option for patients who are blind in the near future. Therefore, the focus should be on increasing the benefits for patients who undergo this therapy. Further advancement of optogenetic tools with improved properties and modified viral vectors (Section 3 and Section 4) is expected to improve the quality of vision provided by optogenetic therapies.

### 2.4. Application for Photoreceptor Transplantation

While basic optogenetic therapy targets the above-mentioned innate retinal cells, there are unique reports of optogenetics applications for photoreceptor transplantation therapy. Garita-Hernandez et al. introduced hyperpolarizing opsins into rod photoreceptor precursors from donor mice and cone photoreceptors from human induced pluripotent stem cells, and confirmed that both optogenetically engineered photoreceptors successfully restored vision after transplantation into blind mice [30,31]. They described that using optogenetically engineered donor cells turns photoreceptor transplantation into RPE-independent therapy, because optogenetic response does not rely on chromophore replenishment. Additionally, this approach could be the only way for optogenetic therapy to maintain near-perfect retinal processing after innate photoreceptor loss.

## 3. Optogenetic Tools

The challenge for visual restoration began with ChR2, which was the first widely used optogenetic tool, extracted from the green alga *Chlamydomonas reinhardtii* [32]. ChR2 is a member of the light-gated ion channels or pumps known as microbial (type 1) opsins. To improve optogenetic therapy for retinal degeneration, engineering of ChR2 or other microbial opsins has been applied in subsequent studies. Based on a simple and robust activity and accumulated evidence from animal experiments, microbial opsin-based optogenetic therapy has been translated into multiple clinical trials. Although microbial opsins carry great potential, these opsins have some drawbacks in restoring human vision, including limited light sensitivity owing to the lack of an amplification system and the immunogenicity of exogenous proteins. Animal (type 2) opsins are light-activated G-protein-coupled receptors (GPCRs) found in higher eukaryotes, including humans. Several classes of animal opsins have been considered as alternative options for optogenetic therapy, with the expectation of superior light sensitivity derived from intracellular signaling cascades. In this section, we briefly explain the currently available optogenetic tools for visual restoration (Table 1).

### 3.1. Microbial (Type 1) Opsins

Depending on the strategies and target cells, we can choose either or both of depolarizing and hyperpolarizing opsins. Basically, depolarizing opsins are used to resemble ON responses of inner retinal cells (bipolar cells and RGCs), and hyperpolarizing opsins are used to target dormant cones. The hyperpolarizing opsins are also applied to directly restore OFF responses of inner retinal cells, which could lead to preserved retinal processing. Combination therapies using depolarizing and hyperpolarizing opsins have been attempted previously [37], although none have reached the clinical stage yet.

#### 3.1.1. Depolarizing Opsin

ChR2 is a non-specific cation channel that can depolarize neurons. Earlier studies have shown that ChR2 expression in RGCs restores light responses in *rd1* mice [8] and RCS rats [33]. The restoration of vision in *rd1* mice was also demonstrated by ChR2 expression in ON bipolar cells [14]. The limitations of ChR2-based therapies include light and wavelength sensitivity. In a behavioral study, intense blue light (2.25 × 10^15^ photon/cm^2^s) was needed to observe visual-guided responses [14,38]. Moreover, visual-evoked potentials (VEP) were not elicited by stimuli over 550 nm [8,33,38]. These outcomes lead not only to an inability to detect red light, but may also cause photoreceptor toxicity after strong blue light exposure, which motivated ChR engineering.

Calcium translocating channelrhodopsin (CatCh) was created as a ChR2 mutant with enhanced Ca^2+^ permeability and 70-fold higher light sensitivity than ChR2 [73]. When CatCh was transduced into RGCs in macaques, reliable electrophysiological responses to light stimulus within the safety limits for the human eye were observed. Although CatCh successfully increases light sensitivity compared with ChR2, more intense light is required to maximize the response [45]. The improved light sensitivity of CatCh is associated with its ability to respond to prolonged light stimulation [74]. While the tradeoff between sensitivity and temporal resolution should be considered, a strategy for prolonging the deactivation or off-rate has been developed. Accordingly, two ChR2 mutants (L132C/T159S) have shown better light sensitivity, which increased by approximately 2 log units compared with wild-type ChR2 [46]. Using the same strategy, CoChR-H94E/L112C/K264T (CoChR-3M) was developed from the mutagenesis of ChR from *Chloromonas oogama* [49]. CoChR-3M was transduced into RGCs in *Opn4^−/−^/Gnat1^−/−^/Cnga3^−/−^* triple-knockout transgenic mice and showed that it could be the first ChR2-mediated optogenetic tool that works under ambient light conditions. In a behavioral study, a visual-guided response of treated transgenic mice was observed at a light intensity as low as 6.4 × 10^12^ photon/cm^2^s with single-wavelength light and 10 μW/cm^2^ with natural white light, while preserving temporal resolution [48]. Chronos (ShChR) is ChR obtained from *Stigeoclonium helveticum* that has faster kinetics than earlier tools with high light sensitivity [49]. Its variant, ChronosFP, has already been translated into a clinical trial (Section 5).

**Figure 3:**
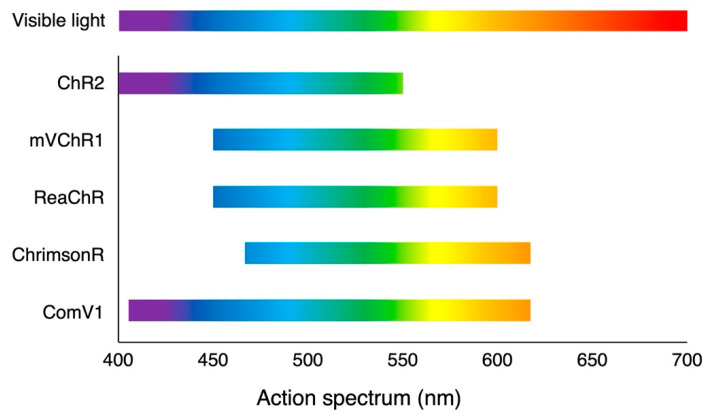
Action spectra of depolarizing opsins. Engineered variants have red-shifted spectra compared with ChR2.

**Figure 4:**
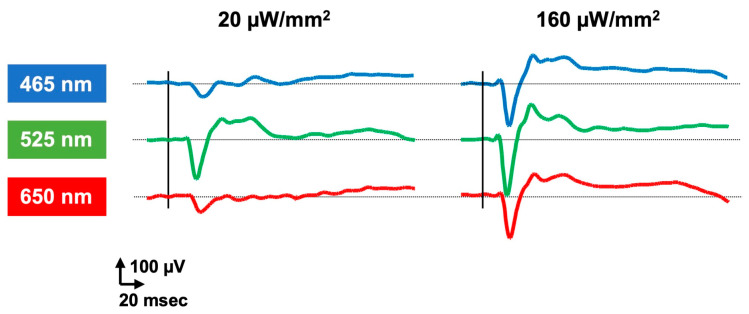
Recordings of visual-evoked potential (VEP) in ComV1-transduced RCS rat. Visual responses to stimuli of broad wavelength ranges within visible light spectrum were demonstrated.

An additional approach involves the use of red-shifted variants (Figure 3). This is a reasonable solution because the safety threshold of light-induced retinal toxicity highly depends on the wavelength of light; that is, red light can lower the risk of retinal damage (ICNIRP, 2013 [75]). The first red-shifted ChR of VChR1 was derived from *Volvox carteri* [76]. Indeed, VChR1 has a red-shifted action spectrum that peaks at 535 nm, but due to poor membrane trafficking in mammals it cannot be used to restore vision [55]. Red-activatable ChR (ReaChR) has then been developed through rational mutagenesis using VChR1 as a template to engineer red-shifted ChR variants, which has improved spectrum responses greater than 600 nm [77]. ReaChR has been demonstrated to function in *rd1* mice, macaques, and human retinal explants [50]. Furthermore, ChrimsonR, the most red-shifted variant to date, was obtained through mutagenesis (K176R) of a naturally found variant (Chrimson) in *Chlamydomonas noctigama* [49]. The spectral peak of ChrimsonR was 45 nm longer than that of the ReaChR. ChrimsonR expression in RGCs has been confirmed to restore light sensitivity in primates [53], which was successfully translated to a subsequent clinical trial [27]. This ChrimsonR-based optogenetic therapy employs an external goggle device [78] that converts the visual scene into light stimulation detectable by the treated retina to compensate for the limited light sensitivity. Modified VChR1 (mVChR1) was generated to improve plasma membrane localization. This variant is a chimera of VChR1 and *Chlamydomonas* channelrhodopsin-1 (ChR1) in which the N-terminal fragment of VChR1 is replaced by that of ChR1. The transduction of mVChR1 into the RGCs in RCS blind rats restored VEP responses to a stimulus with a wide range of 450–600 nm [55], and the follow-up study confirmed its stability over a year [56]. Nevertheless, its drawback is the requirement of high bright light intensity to elicit responses. Further engineering using a bioinformatics approach has led to the development of ex3mV1Co (ComV1). This variant of mVChR1 has nucleotide sequences encoding two sites in the extracellular loops (ex1, ex3) replaced by homologous sequences of ChR1 or ChR2, and the transmembrane of the sixth replaced by that of CoChR. In RCS rats, transduction of ComV1 into the RGCs demonstrated visual responses to light with a wavelength of 405–617 nm, and the threshold response to light was appropriately 1.0 × 10^13^ photon/cm^2^s [59] (Figure 4). Thus, ComV1 has the potential to restore daylight vision without an external device.

Multicharacteristic opsin (MCO1) was developed by Nanoscope Technologies LLC as an ambient light-activatable optogenetic tool. Although the molecular details of MCO1 have not been completely provided in the literature, viral expression of MCO1 in bipolar cells has demonstrated the recovery of visually-guided behavior with ambient light in *rd10* mice [60,79] and safety in dogs [61].

#### 3.1.2. Hyperpolarizing Opsin

NpHR, is a light-gated chloride pump with a hyperpolarizing effect. To diminish the cellular toxicity induced by aggregate formation, enhanced NpHR (eNpHR) was engineered [80]. eNpHR expression in the remaining cones in RP mouse models has been shown to restore visual responses with functional retinal circuits [13].

Red-shifted hyperpolarizing opsins that are preferred for clinical use have been explored. Halo57, derived from *H. salinarum,* was identified as having a red-shifted spectrum with robust light activity, and further engineering resulted in the development of Jaws [63]. In the *rd1* mouse retina, the expression of Jaws has been shown to have higher light sensitivity to light with broad wavelengths (470, 550, and 600 nm) compared with eNpHR and wild-type Halo57 [63]. Restoration of cone function through transduction of Jaws has been confirmed in macaques (in vivo) and human retinas (postmortem explants and induced pluripotent stem cell-derived retinal organoids) [64]. Thus far, Jaws has been a substantial tool for potential cone rescue therapy.

### 3.2. Animal (Type 2) Opsins

#### 3.2.1. Melanopsin

Melanopsin, a native photopigment of the retina expressed in a particular population of RGCs, namely intrinsically photosensitive RGCs, is an animal opsin first applied to optogenetic therapy. Although the original role of melanopsin is associated with the regulation of non-visual functions, such as pupillary response and circadian entrainment [81,82], it is expected to be a candidate tool for visual restoration [83]. Using an optogenetic approach, ectopic expression of melanopsin in non-selective RGCs has been demonstrated to restore visual function in *rd1* blind mice [65]. The positive aspects of using melanopsin include not only its ability to regenerate chromophores by itself, but also the supposed ubiquity of the required signaling cascade [65]. Indeed, a later study showed that melanopsin expression in bipolar cells following subretinal injection restored visual function in *rd1* mice [68]. An obvious limitation is the slow response to light, which restricts the temporal resolution. Melanopsin has also been used as a component of the unique chimeric optogenetic tool Opto-mGluR6. Opto-mGluR6 consists of a light-sensitive domain of melanopsin and an intracellular domain of the ON bipolar cell-specific GPCR mGluR6. This chimeric tool enables the use of intracellular effectors of innate ON bipolar cells, which compensates for the slow kinetics of melanopsin. Recovery of vision through Opto-mGluR6 expression in ON bipolar cells has been confirmed in *rd1* mice [18].

#### 3.2.2. Rhodopsin

Rhodopsin is a key photosensitive protein of native phototransduction in humans and could be a candidate tool for optogenetic therapy. It is well known to have extremely good light sensitivity that can react with a single photon, owing to the sophisticated G-protein-coupled signaling cascade [84]. When human rhodopsin was expressed in ON bipolar cells in blind *rd1* mice, relatively high light sensitivity was observed with improved kinetics compared to that of melanopsin [23,69]. Unexpectedly, light responses were observed to be as low as ~10^12^ photon/cm^2^s [23], and the sensitivity was equivalent to that of melanopsin. Rhodopsin is densely packed in the specialized structure of the disc membrane of rod photoreceptors. Since the ectopic expression of rhodopsin in non-photoreceptor cells must lead to a lower intracellular density compared to that in photoreceptors, it is reasonable that the ability to respond to light is deteriorated. The signaling cascades employed by cells with ectopic rhodopsin expression, which also affects light sensitivity, remain unclear. Moreover, another obvious concern is that rhodopsin essentially requires recycling chromophores of 11-*cis*-retinal, which requires the cooperation of the retinoid cycle involving RPEs [85], although it has been demonstrated that ectopic expression of human rhodopsin restores visual function in in vivo rodent assays without exogenous augmentation of the chromophore [23].

*Gloeobacter* and human chimeric rhodopsin (GHCR) were developed to overcome this limitation, where the second and third intracellular loops of *Gloeobacter* rhodopsin were replaced with human rhodopsin, in addition to the E132Q mutation [86]. This chimeric rhodopsin is theoretically independent of 11-*cis*-retinal regeneration, and its expression in *rd1* blind mouse retinas has shown visual restoration with relatively high light sensitivity (10^13^ photon/cm^2^s) [72].

#### 3.2.3. Cone Opsin

Recent studies have applied cone opsins to optogenetic therapy. Transduction of medium-wavelength cone opsin (MW-opsin) into RGCs restored *rd1* mouse vision and was suggested to have faster light response than rhodopsin [70]. Targeting MW-opsin in bipolar cells could also be effective in *rd1* mice [20].

Finally, there is an interesting report investigating the utility of chimeric human opsins, wherein chimeras consisting of intracellular domains of melanopsin and light-sensitive domains of rhodopsin or long-wavelength cone opsin were generated to exploit the supposed ubiquitous signaling cascade of melanopsin. Unfortunately, these chimeras did not work as well as wild-type human opsins [87].

## 4. Gene Delivery

### 4.1. Adeno-Associated Viral Vectors

Adeno-associated virus (AAV) is a small, non-enveloped, non-pathogenic virus that packages a 4.7-kb single-stranded DNA genome [88]. Currently, AAV vectors are the most commonly employed tool for retinal gene therapy based on their apparent safety and transduction efficiency to the retina. While many serotypes of AAV with variable tropisms are available, the clinical efficacy of AAV2 vectors has been shown in previous retinal gene replacement therapies [89,90,91,92]. Early studies on optogenetic therapy have also employed AAV2 to transduce optogenetic tools to RGCs through intravitreal injection [8,33,65]. In combination with AAV2, ubiquitous promoters such as the hybrid cytomegalovirus (CMV) enhancer/chicken β-actin (CAG) promoter and CMV promoter have been used in many studies. One study reported that the human gamma-synuclein gene (SNCG) promoter showed superior CatCh expression in RGCs compared to the CMV promoter in both *rd1* mice and macaques [45].

Then, the modified vectors obtained from AAV engineering were incorporated into subsequent studies. Tyrosine-mutated AAVs are generated through rational mutagenesis to improve intracellular nuclear trafficking, based on the finding that phosphorylation of surface-exposed tyrosine residues on AAV leads to ubiquitination and subsequent proteasome-mediated degradation. Intravitreal injection of AAV2 quadruple mutants (Y272, 444, 500, 730F; 4YF) has been demonstrated to transduce bipolar cells using either the Grm6 [16,20,72] or L7-5 promoter [24,51] in rodent models. When AAV2(4YF) was used with the hSyn-1 promoter, optogenetic gene expression was dominant in RGCs [70]. Another strategy of AAV engineering is directed evolution, which consists of the iterative generation of diverse viral libraries and selection of variants with the desired properties. AAV2(7m8), which has a 10-amino acid insertion in loop IV of AAV2, was discovered using this directed evolution approach in mice. This mutant was originally used to enhance retinal penetration to transduce photoreceptors from the vitreous side [93]. It has been successfully applied in optogenetic therapy, and is expected to target bipolar cells (with the Grm6 promoter) in *rd1* mice [17,20]. AAV2(7m8) has also been employed in RGC-targeted optogenetic therapy, which is expected to improve retinal transduction efficiency in non-human primates [53], followed by a clinical trial with the same drug context [27] (Section 5). Moreover, a combination of tyrosine mutation (AAV[4YF+TV]) and 7m8 peptide insertion (AAV[7m8]) in a single AAV2 (called AAV2[MAX]) [94] has demonstrated improved retinal transduction in mice compared to each of the two independent mutants. Recently, a new AAV2-based variant (R100) was discovered through directed evolution in macaques. R100 has the ability to transduce relevant cells in the primate retina through intravitreal injection [95]. To the best of our knowledge, neither AAV(MAX) nor R100 have currently been applied in optogenetic therapy. Hopefully, these new-generation AAV vectors with better transduction efficiency enable the conversion of a larger population of retinal cells into artificial photoreceptors and can be expected to provide higher quality vision to patients. As regards targeting deeper retinal cells, including bipolar cells and dormant cones, AAV8 [63,66] and its mutant (Y733F) [15,68] or AAV9 [61,62], administered through subretinal injection, have been used as other options.

### 4.2. Intervention

For retinal gene therapy, subretinal and intravitreal injections are the major options for delivering therapeutic genes. In principle, an injection route proximal to the target cell would be advantageous. Therefore, gene replacement therapy prefers subretinal injection to target photoreceptors or RPEs [5]. While subretinal injection enables the delivery of genes to the outer retina at the expected concentration, its drawbacks include spatially limited efficiency and induced retinal damage. Early experiences warned that the necessity of iatrogenic detachment of the vulnerable retina could lead to retinal thinning [96]. Intravitreal injection is less invasive and can theoretically treat the entire retina. A representative challenge is how the drugs will pass through physical barriers, including the vitreous and inner limiting membranes [97]. Internal limiting membrane disruption by enzyme digestion [98,99] or surgical peeling [100] is worth exploring to improve treatment efficacy. Considering that the injected solution is diluted by the vitreous humor, another issue is the requirement of a relatively high number of viral vectors, which could increase the risk of inflammatory reactions. Recent developments in viral vectors with improved gene transduction efficiency [93,94,95] or fewer immunogenic features [101] may contribute to the circumvention of harmful inflammatory responses.

In the optogenetic field, intravitreal injection is preferred because the inner retina is the main target. RGC-targeted optogenetic therapy is compatible with intravitreal injections. While bipolar cells could be targeted through intravitreal injection with engineered AAV vectors [16,17,20,24,51,69], some researchers have reported successful transduction of bipolar cells through subretinal injection using AAV2(7m8) [20], AAV8(BP2) [20], or AAV8(Y733F) [15] with the Grm6 ON-bipolar cell promoter in RP mouse models. In contrast, subretinal injections have been used to rescue dormant cones [13,62,63].

## 5. Road to Clinical Application

Currently, five clinical trials on optogenetic therapy are ongoing (Table 2). Four prior studies included patients with retinitis pigmentosa, and one study (NCT05417126) for Stargardt disease was newly registered. The STARLIGHT study used MCO1, similar to the phase 2b RESTORE study for retinitis pigmentosa (NCT04945772). All ongoing clinical trials have adopted intravitreal injections to deliver drugs. AAV serotype 2 is preferred, and its variant AAV2(7m8) was used in the PIONEER study (NCT03326336). The RESTORE and STARLIGHT studies (MCO1-based therapy) plan to target bipolar cells, while other studies target RGCs. Although microbial opsin-based therapies are currently at the forefront, animal opsin-based therapies have been steadily progressing. Recently, a worldwide multicenter team registered the EyeConic study (NCT05294978), which aimed to estimate the prevalence of patients with dormant cones to prepare for the clinical trial of cone opsin-based optogenetic therapy. Some companies are preparing clinical trials using human rhodopsin [23] (Kubota Vision Inc.), CoChR variant [48] (Ray Therapeutics), or chimeric rhodopsin (GCHR) [72] (Restore Vision Inc.). Furthermore, MW-opsin-based therapy [70] (Vedere Bio) and Opto-mGluR6-based therapy [18] (Arctos Medical) were both acquired by Novartis and are expected to lead to new clinical trials.

To develop optogenetic therapy, there are various options for treatment elements, including target cells, optogenetic tools, and gene delivery methods, as presented in this review. Note that the treatment choice mainly depends on the relative advantages and disadvantages, because few preclinical studies have directly compared different options under the same conditions [24,51]. Further comparative studies are warranted, and the best choice for each element should be explored. Continued improvements in optogenetic tools and gene delivery systems are expected. The low light sensitivity of optogenetic tools is being addressed, and next-generation optogenetic tools will have the potential to restore daylight vision. Still, even if an excellent optogenetic tool is available, there is another important concern: virally mediated gene transduction efficiency is lower in primate retinas than in the small animals commonly used in preclinical studies [93,102]. Recently developed vectors that are designed to overcome this issue could be a promising choice, and preclinical studies of optogenetic therapy should involve non-human primates. We should also explore the best combination of the elements. Advancements in each element composing optogenetic therapy could be enhanced by selecting the best combination. Cross-disciplinary approaches with understanding of relevant technologies will be helpful in future research.

Shortly thereafter, the results of preceding clinical trials will provide important insights into the outcomes of optogenetic therapy in human patients. How much vision could be restored by this therapy? The outcome assessment may require some ingenuities, in addition to conventional methods, to detect the slight changes of visual function in patients with low vision. In the preceding case report, detailed visually guided behavioral tests in combination with electrophysiological examinations were used to show the partial recovery of vision [27]. Full-field stimulus testing, which has been established as a specialized visual assay for low-vision patients with retinal degeneration, could be a useful additional choice [103]. Patient selection will also become an important topic. This novel optogenetic therapy is expected to be open to every patient with late-stage retinal degeneration; however, the extent of retinal remodeling may be key to post-treatment vision. Precise evaluation of the inner retinal condition should be performed before treatment; to this end, chromatic pupillometry or electrically evoked phosphene thresholds are available [104,105]. Indeed, the urgent tasks are confirming the reproducibility of the results of visual restoration and the safety of treatment in human patients, but the next challenge would be improving the quality of post-treatment vision. It is expected that, based on the clinical experiences with human patients, future reverse translational research will indicate the direction that we should follow to overcome the limitations of using currently available options. We hope that continuous advancements in optogenetic therapy will provide better restored vision to patients with retinal degenerative diseases.

## Figures and Tables

**Table 1:** Optogenetic tools for visual restoration.

	Rodents	Dogs	Primates	Clinical Trials
**Microbial Opsins**				
Depolarizing opsins				
ChR2	Bi et al. [8]; Tomita et al. [33]; Farah et al. [34]; Lagali et al. [14]; Tomita et al. [35]; Ivanova and Pan [36]; Zhang et al. [37]; Tomita et al. [38]; Thyagarajan et al. [39]; Ivanova et al. [40]; Doroudchi et al. [15]; Sugano et al. [41]; Isago et al. [42]; Wu et al. [43]	-	Ivanova et al. [44]	NCT02556736
Enhanced light sensitivity variants				
CatCh	Chaffiol et al. [45]	-	Chaffiol et al. [45]	-
ChR2(L132C/T159S)	Pan et al. [46]; Lu et al. [47]	-	-	-
CoChR-3M	Ganjawala et al. [48]; Wright et al. [21]	-	-	-
ChronosFP	Klapoetke et al. [49]	-	-	NCT04278131
Red-shifted variants				
ReaChR	Sengupta et al. [50]; Wright et al. [21]; Gilhooley et al. [24]	-	Sengupta et al. [50] (ex vivo)	-
ChrimsonR	Cheong et al. [51]	-	McGregor et al. [52]; Gauvain et al. [53]; McGregor et al. [54]	NCT03326336 (PIONEER)
mVChR1	Tomita et al. [55]; Sugano et al. [56]; Sato et al. [57]; Tabata et al. [58]	-	-	-
ComV1	Watanabe et al. [59]	-	-	-
MCO1	Wright et al. [60]	Tchedre et al. [61]	-	NCT04919473, NCT04945772 (RESTORE), NCT05417126 (STARLIGHT)
Hyperpolarizing opsins				
eNpHR	Busskamp et al. [13]	Nikonov et al. [62]	-	-
Jaws	Chuong et al. [63]; Khabou et al. [64]	-	Khabou et al. [64]	-
**Animal opsins**				
Melanopsin	Lin et al. [65]; Liu et al. [66]; Ameline et al. [67]; De Silva et al. [68]; Gilhooley et al. [24]	-	-	-
Rhodopsin	Cehajic-Kapetanovic et al. [23]; Gaub et al. [69]; Eleftheriou et al. [19]; Berry et al. [70]; McClements et al. [20]; Wright et al. [21]	-	-	-
Cone opsin	Berry et al. [70]; McClements et al. [20]	-	-	-
Chimeras				
Opto-mGluR	van Wyk et al. [18]; Schilardi et al. [71]	-	-	-
GHCR	Katada et al. [72]	-	-	-

**Table 2:** Ongoing clinical trials of optogenetic therapy.

	Phase	Patients	Actual Study Start Date	Drug	Tool	Vector	Intervention	Sponsor	Reported results
NCT02556736	I/IIa	retinitis pigmentosa	14 December 2015	RST-001	ChR2	AAV2	Intravitreal injection	AbbVie, Chicago, IL, USA (Allergan Inc., Irvine, CA, USA)	Clinical trials.gov. (https://clinicaltrials.gov/ct2/show/results/NCT02556736 accessed on 29 September 2022.)
NCT03326336 (PIONEER)	I/IIa	retinitis pigmentosa	26 September 2018	GS030-DP/ GS030-MD	ChrimsonR	AAV2(7m8)	Intravitreal injection	GenSight Biologics, Paris, France	Sahel et al. [27]
NCT04919473, NCT04945772 (RESTORE)	I/IIa, b	retinitis pigmentosa	23 October 2019, 13 July 2021	vMCO-010	MCO	AAV2	Intravitreal injection	Nanoscope Therapeutics Inc., Dallas, TX, USA	Press release (https://www.ophthalmologytimes.com/view/optogenetic-gene-therapy-restores-vision-in-11-rp-patients accessed on 29 September 2022.)
NCT04278131	I/II	retinitis pigmentosa	6 February 2020	BS01	ChronosFP	AAV (serotype is undisclosed)	Intravitreal injection	BionicSight LLC, New York, NY, USA	Press release (https://www.globenewswire.com/news-release/2021/03/30/2201412/0/en/First-Four-Patients-In-Bionic-Sight-s-Optogenetic-Gene-Therapy-Trial-Are-Able-To-Detect-Light-And-Motion.html accessed on 29 September 2022.)
NCT05417126 (STARLIGHT)	IIa	Stargardt disease	5 July 2022	vMCO-010	MCO	AAV2	Intravitreal injection	Nanoscope Therapeutics Inc., Dallas, TX, USA	Not reported

## Data Availability

Not applicable.
